# Thio-barbiturate-derived compounds are novel antioxidants to prevent LPS-induced inflammation in the liver

**DOI:** 10.18632/oncotarget.21714

**Published:** 2017-10-10

**Authors:** Kyoung Mi Moon, Bonggi Lee, Ji Won Jeong, Dae Hyun Kim, Yun Jung Park, Hye Rim Kim, Ji Young Park, Min Jo Kim, Hye Jin An, Eun Kyeong Lee, Young Mi Ha, Eunok Im, Pusoon Chun, Jin Yeul Ma, Won-Kyung Cho, Hyung Ryong Moon, Hae Young Chung

**Affiliations:** ^1^ Molecular Inflammation Research Center for Aging Intervention, College of Pharmacy, Pusan National University, Busan, Korea; ^2^ Laboratory of Medicinal Chemistry, College of Pharmacy, Pusan National University, Busan, Republic of Korea; ^3^ Department of Chemistry, Dong-A University, Busan, Republic of Korea; ^4^ College of Pharmacy, Inje University, Inje-ro, Gyeongnam, Korea; ^5^ Korean Medicine-Application Center, Korea Institute of Oriental Medicine, Daegu, Republic of Korea

**Keywords:** oxidative stress, antioxidant, compound 2d, compound 2l, inflammation

## Abstract

Liver inflammation is closely associated with metabolic syndrome. Oxidative stress plays a synergistic role in inflammation by activating nuclear factor kappa B (NF-κB) signaling in the liver. Therefore, substantial efforts have been made to develop compounds that inhibit the generation of oxidative stress and activation of NF-κB. We synthesized twenty-six novel 5-(substituted benzyl)-2-oxo- and 5-(substituted benzyl)-2-thioxo-dihydropyrimidine-4,6(1*H*,5*H*)-dione derivatives for the development of potential antioxidants and examined their biological activities *in vitro* and *in vivo*. Thio-barbiturate-derived compounds 5-[4-hydroxy-3-methoxybenzy]-2-thioxodihydropyrimidine-4,6[1*H*,5*H*]-dione (2d) and 5-[4-hydroxy-3,5-methoxybenzy]-2-thioxodihydropyrimidine-4,6[1*H*,5*H*]-dione (2l) had the strongest inhibitory effect on reactive oxygen species and peroxynitrite generation *in vitro*. Furthermore, oral administration of compounds 2d and 2l in mice notably suppressed lipopolysaccharide (LPS)-induced oxidative stress and NF-κB activation in the liver. Because macrophages play an essential role in liver inflammation, we investigated the effects of these compounds on inflammatory signaling in LPS-induced RAW264.7 macrophages. LPS-induced NF-κB activation and protein expression of cyclooxygenase 2 and inducible nitric oxide synthase were inhibited by pretreatment of these compounds in macrophages. In parallel with this finding, the phosphatase and tensin homolog deleted on chromosome 10 (PTEN) and AKT signalings, which are upstream activators of p65, were decreased by these compounds in macrophages. Our study suggests that compounds 2d and 2l inhibit oxidative stress and NF-кB-mediated inflammation, at least partially, through suppressing PTEN/AKT signaling. Therefore, these compounds may be useful as therapeutic agents for the amelioration of inflammatory diseases.

## INTRODUCTION

Lipopolysaccharide (LPS) induces secretion of pro-inflammatory cytokines by activating macrophages, which play a causal role in acute and chronic inflammation [1]. As liver inflammation is closely associated with hepatic cirrhosis, liver lesion, hepatic fibrosis, and liver cancer, treatment of inflammation is an important strategy for decreasing them [2–4]. Macrophages are a major immune cell population in the liver and play an essential role in liver inflammation-related diseases [1, 5]. Macrophages can be activated in response to various inflammatory stimuli, including LPS [6–8].

The phosphatase and tensin homolog deleted on chromosome 10 (PTEN) is a widely expressed protein phosphatase [9]. Since various studies have reported that PTEN is frequently mutated or deleted in a variety of human cancers, PTEN has been considered as a tumor suppressor gene [9, 10]. In addition, PTEN is associated with reactive oxygen species (ROS)-mediated inflammatory signaling [11]. ROS increases autophosphorylation of PTEN and thereby inhibits its phosphates activity. As a result, activated AKT stimulates nuclear factor kappa B (NF-κB)-mediated inflammation [12].

Basally, inactive NF-κB is localized to the cytoplasm after forming a complex with an inhibitor of kappa B (IκB), an inhibitory factor of NF-κB [13, 14]. Upon stimulation, activated AKT phosphorylates and activates inhibitor of kappa B kinase (IKK), which phosphorylates IκB [15]. Then, phosphorylated IκB is dissociated from NF-κB, and activated NF-κB is translocated to the nucleus, where it interacts with specific DNA binding sites to induce inflammatory gene transcription including, inducible nitric oxide synthase and cyclooxygenase 2 [16, 17].

Because there is a close relationship between oxidative stress and inflammation, we synthesized novel compounds with antioxidant activity. Of these compounds, we screened 5-[4-hydroxy-3-methoxybenzy]-2-thioxodihydropyrimidine-4,6[1*H*,5*H*]-dione (2d) and 5-[4-hydroxy-3,5-methoxybenzy]-2-thioxodihydropyrimidine-4,6[1*H*,5*H*]-dione (2l) and further studied their anti-inflammatory properties and underlying mechanisms using C57BL/6 mice and RAW264.7 macrophages after LPS treatment.

## RESULTS AND DISCUSSION

### Screening of compounds 2d and 2l as ROS and ONOO^-^ scavengers

**Figure 1:**
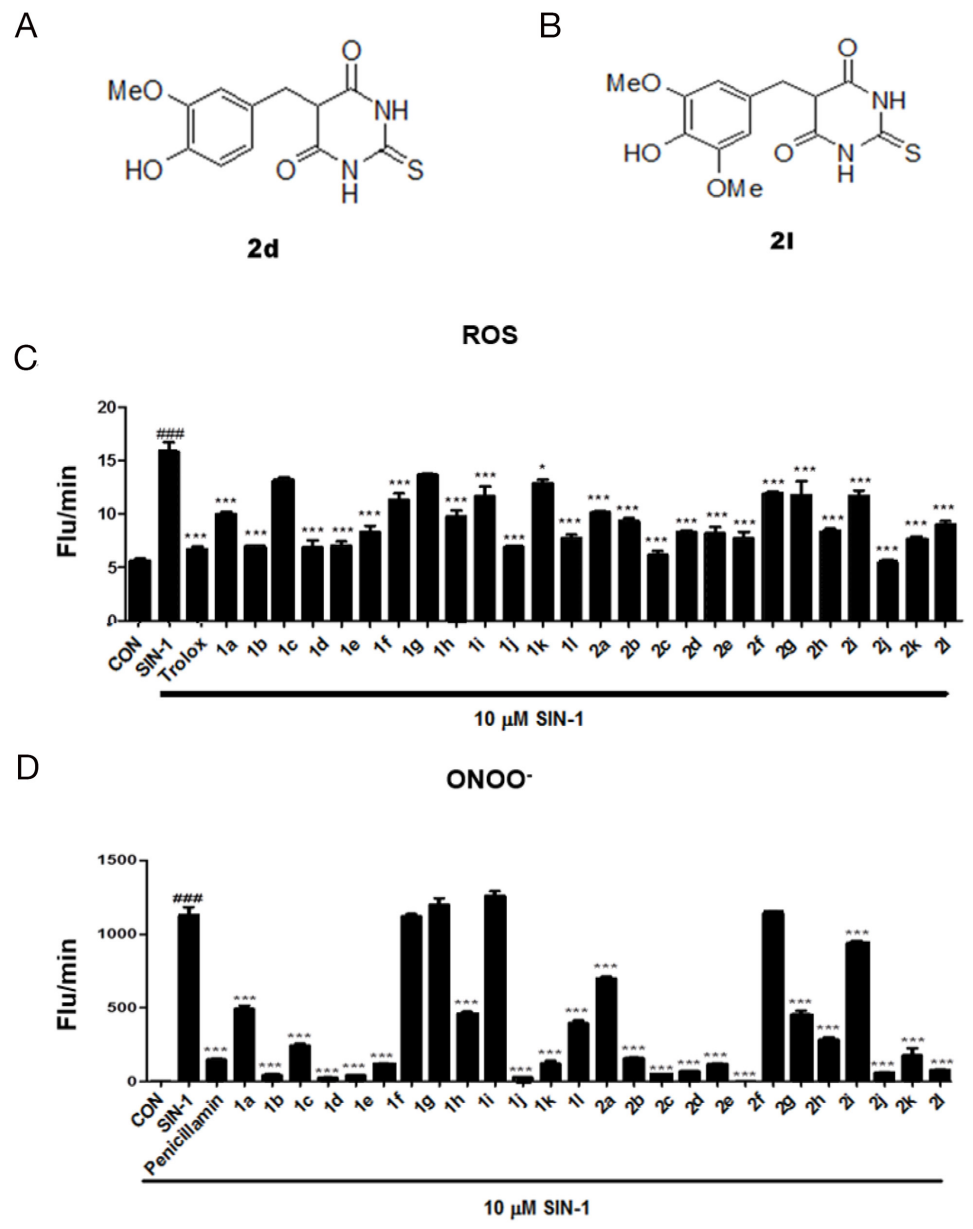
Screening of thio-barbiturate-derived compounds **(A)** The structures of 5-[4-hydroxy-3-methoxybenzy]-2-thioxodihydropyrimidine-4,6[1*H*,5*H*]-dione (2d) and **(B)** 5-[4-hydroxy-3,5-methoxybenzy]-2-thioxodihydropyrimidine-4,6[1*H*,5*H*]-dione (2l). **(C)** Reactive oxygen species (ROS) and **(D)** peroxynitrite (ONOO^-^) were measured *in vitro* using DCFDA and DHR123, respectively. The data are shown as the mean ± SEM (n=3). Statistical results of one-way ANOVA, followed by the Dunnett’s test: *###p* < 0.001 vs. a control group, ^*^p < 0.05, ^***^p < 0.001 vs. the 3-morpholinosydnonimine (SIN-1)-treated group.

**Figure 2:**
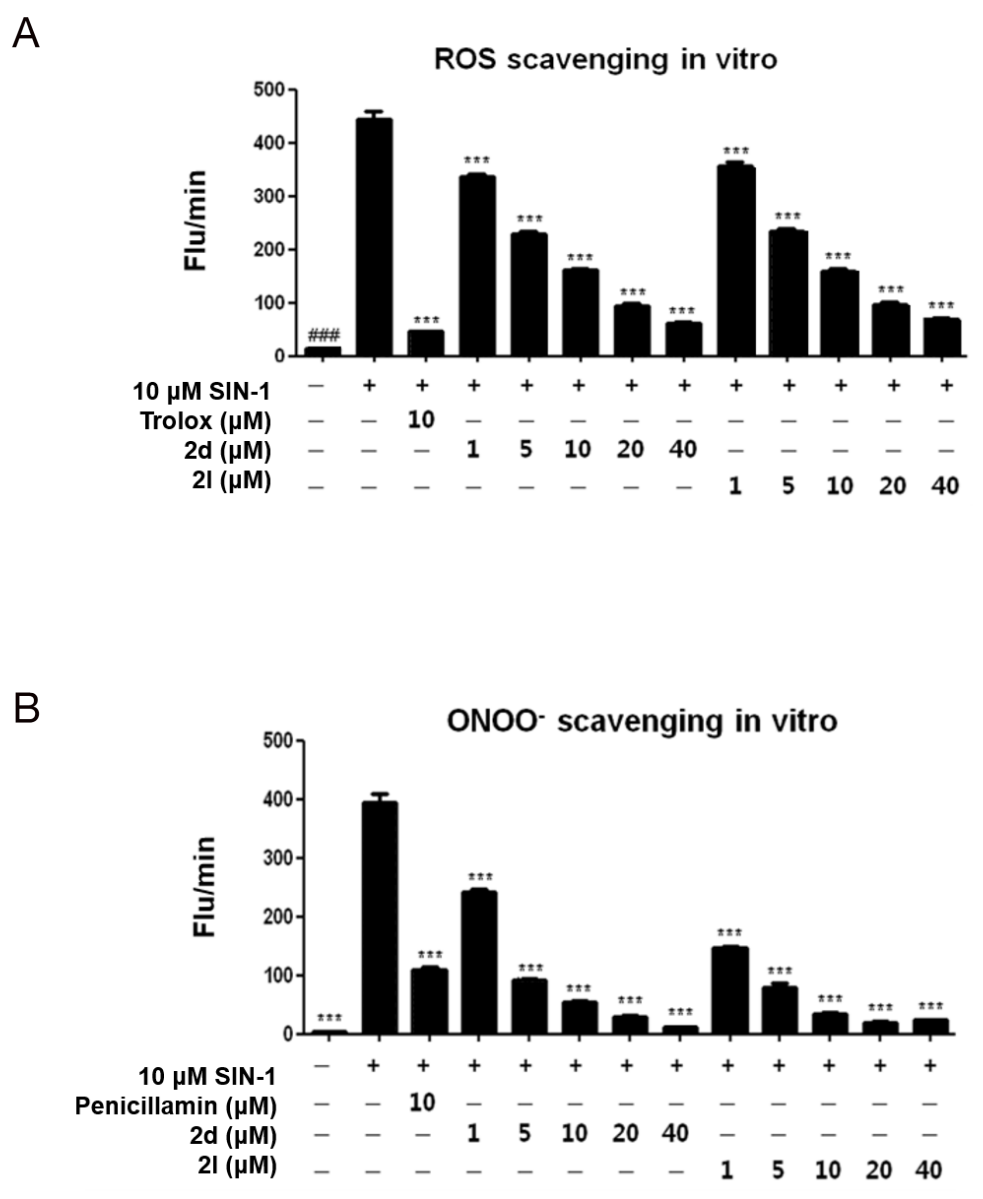
Effects of compound 2d or compound 2l treatment on ROS and ONOO^-^ scavenging *in vitro* **(A)** ROS and **(B)** ONOO^-^ were measured *in vitro* using DCFDA and DHR123, respectively. Trolox and penicillamine were used as a known ROS and ONOO^-^ inhibitor, respectively. The data are shown as the mean ± SEM (n=5). Statistical results of one-way ANOVA, followed by the Dunnett’s test: *###p* < 0.001 vs. a control group, ^***^p < 0.001 vs. a SIN-1-treated group.

Reactive oxygen species (ROS) and peroxynitrite (ONOO^-^) play a critical role in liver inflammation by regulating the expression of pro-inflammatory cytokines [18]. We examined the effects of 26 5-(substituted benzyl)-2-oxo- and 5-(substituted benzyl)-2-thioxo-dihydropyrimidine-4,6(1*H*,5*H*)-dione derivatives (Table 1) on ROS and ONOO^-^ production *in vitro* after 3-morpholinosydnonimine (SIN-1) stimulation. The well-known ROS inhibitor trolox and the ONOO^-^ inhibitor penicillamine were used as positive controls (Figure 1C-1D). Of these derivatives, compounds 2d and 2l were screened further because their ROS and ONOO^-^ scavenging activities were greater than those of the positive controls (Figure 2A-2B). We examined the dose-dependent effects of these compounds on ROS and ONOO^-^ scavenging activity and found that compounds 2d and 2l dose- dependently inhibited SIN-1-induced ROS and ONOO^-^ generation *in vitro* (Figure 2A-2B), suggesting that these compounds inhibit SIN-1-mediatied oxidative stress.

**Table 1:** 5-(Substituted benzyl)-2-oxo- and 5-(substituted benzyl)-2-thioxo-dihydropyrimidine-4,6(1H,5H)-dione derivatives

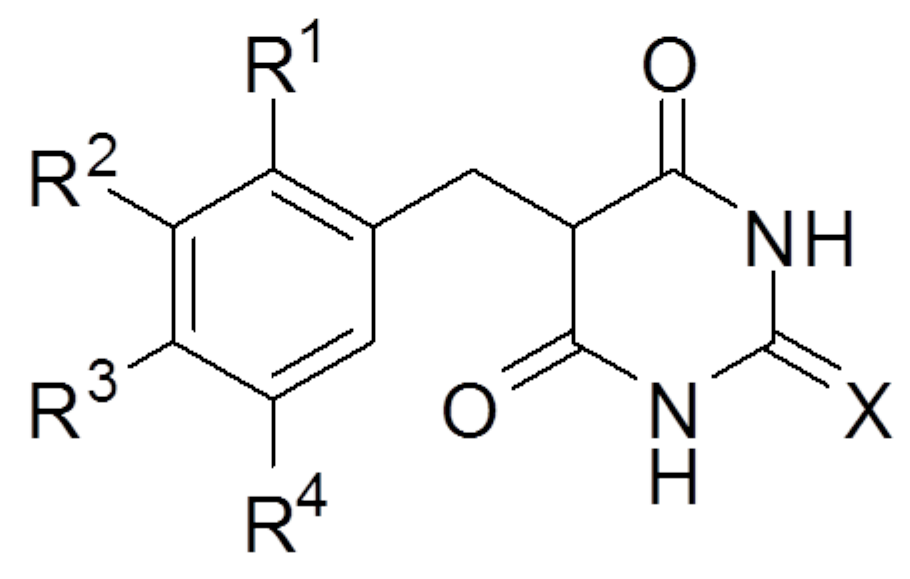					
Compounds	X	R^1^	R^2^	R^3^	R^4^
1a	O	H	H	OH	H
1b	O	H	OH	OH	H
1c	O	OH	H	OH	H
1d	O	H	OMe	OH	H
1e	O	H	OEt	OH	H
1f	O	H	OH	OMe	H
1g	O	H	H	OMe	H
1h	O	H	OMe	OMe	H
1i	O	OMe	H	OMe	H
1j	O	H	OMe	OMe	OMe
1k	O	H	OMe	OH	OMe
1l	O	H	Br	OH	Br
2a	S	H	H	OH	H
2b	S	H	OH	OH	H
2c	S	OH	H	OH	H
2d	S	H	OMe	OH	H
2e	S	H	OEt	OH	H
2f	S	H	OH	OMe	H
2g	S	H	H	OMe	H
2h	S	H	OMe	OMe	H
2i	S	OMe	H	OMe	H
2j	S	OH	H	H	H
2k	S	H	OMe	OMe	OMe
2l	S	H	OMe	OH	OMe
2m	S	H	Br	OH	H
2n	S	H	Br	OH	Br

### Inhibitory effects of compounds 2d and 2l on oxidative stress and NF-κB signaling in the liver

**Figure 3:**
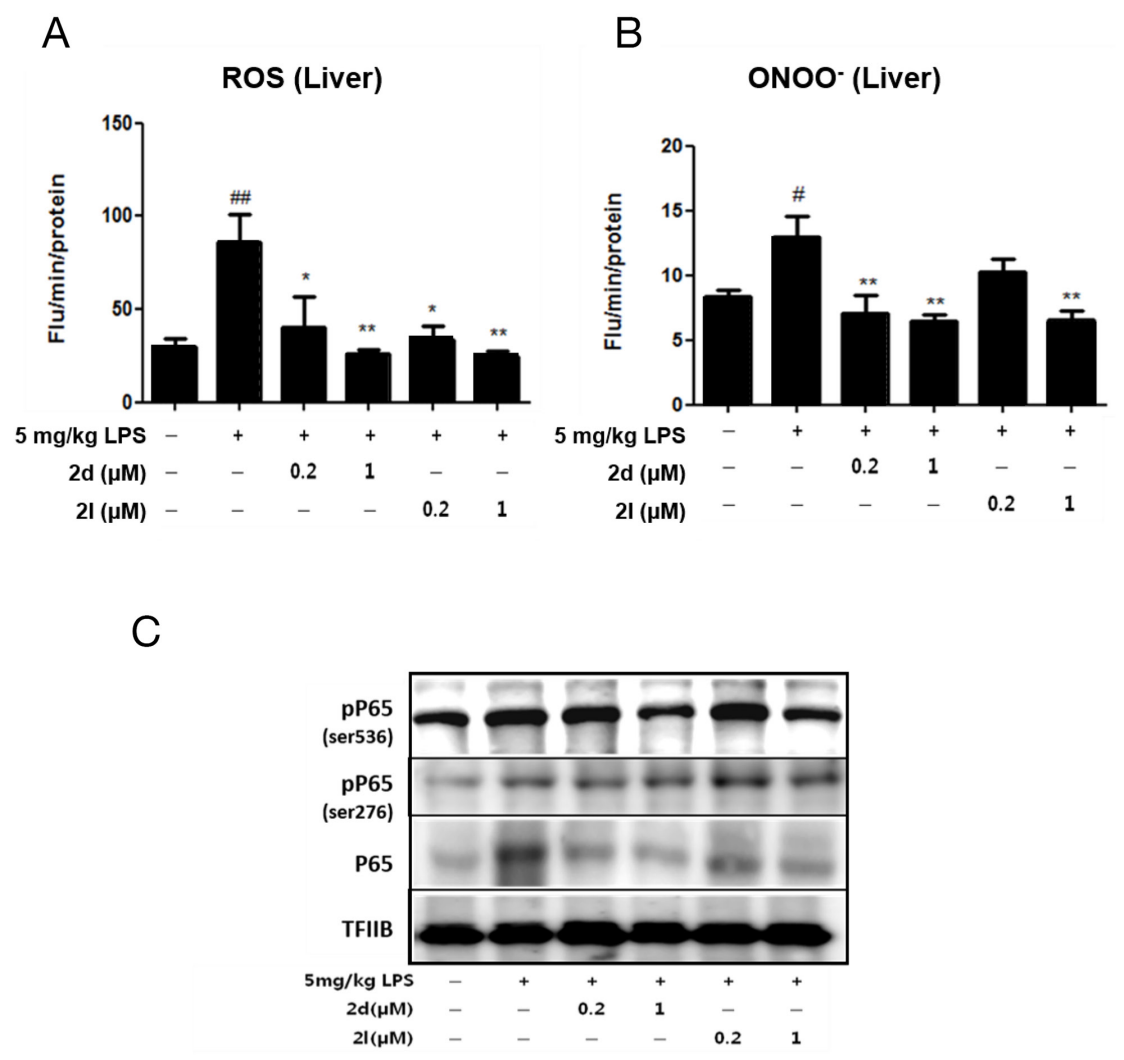
Compounds 2d and 2l ameliorate inflammation in lipopolysaccharide (LPS)-induced liver injury Different concentrations of compounds 2d and 2l (0.2 μM or 1 μM) were orally administered (PO) to C57BL/6 mice, and 2 h later, mice were treated with LPS (5mg/kg). Mice were sacrificed 1h after the LPS injection (n=5/group). Inhibitory effects of compounds 2d and 2l on **(A)** ROS levels and **(B)** ONOO^-^ levels. **(C)** Western blotting was performed to examine the effects of compounds 2d and 2l on p-p65 (ser536), and p65 in the nucleus of the liver. Transcription Factor II B (TFIIB) was used as a loading control for the nucleus. The data are shown as the mean ± SEM. Statistical results of one-way ANOVA followed by the Dunnett’s test : *#p* < 0.05, *##p* < 0.01 vs. a control mice, ^*^p < 0.05, ^**^p < 0.01 vs. LPS-treated mice.

To investigate whether compounds 2d and 2l affect antioxidant and anti-inflammatory signaling in the liver, each compound 2d and 2l (0.2 μM and 1 μM) was delivered to C57BL/6 mice by oral gavage prior to an intraperitoneal injection of lipopolysaccharide (LPS) (5 mg/kg). We examined oxidative stress and its downstream nuclear factor kappa B (NF-κB) activity. ONOO^-^ levels were significantly increased in the LPS-injected group compared to the non-injected group, but pretreatment with compounds 2d and 2l dose-dependently inhibited LPS-induced increases in ROS and ONOO^-^ levels in the liver (Figure 3A-3B), indicating that these compounds inhibited LPS-induced oxidative stress. Because oxidative stress is closely associated with LPS-induced activation of NF-κB signaling, we studied whether the compounds affect NF-κB protein expression and activity. Compounds 2d and 2l at 1μM significantly decreased protein levels of p65 and phosphorylated p65 (Ser536) in the nucleus of the liver (Figure 3C), indicating that the compounds inhibited NF-κB signaling. Considering the close relationship between oxidative stress and NF-κB activation, compound 2d- and compound 2l-mediated inhibition of NF- κB may be associated with the suppression of oxidative stress.

### Inhibitory effects of compounds 2d and 2l on LPS-induced oxidative stress in macrophages

**Figure 4:**
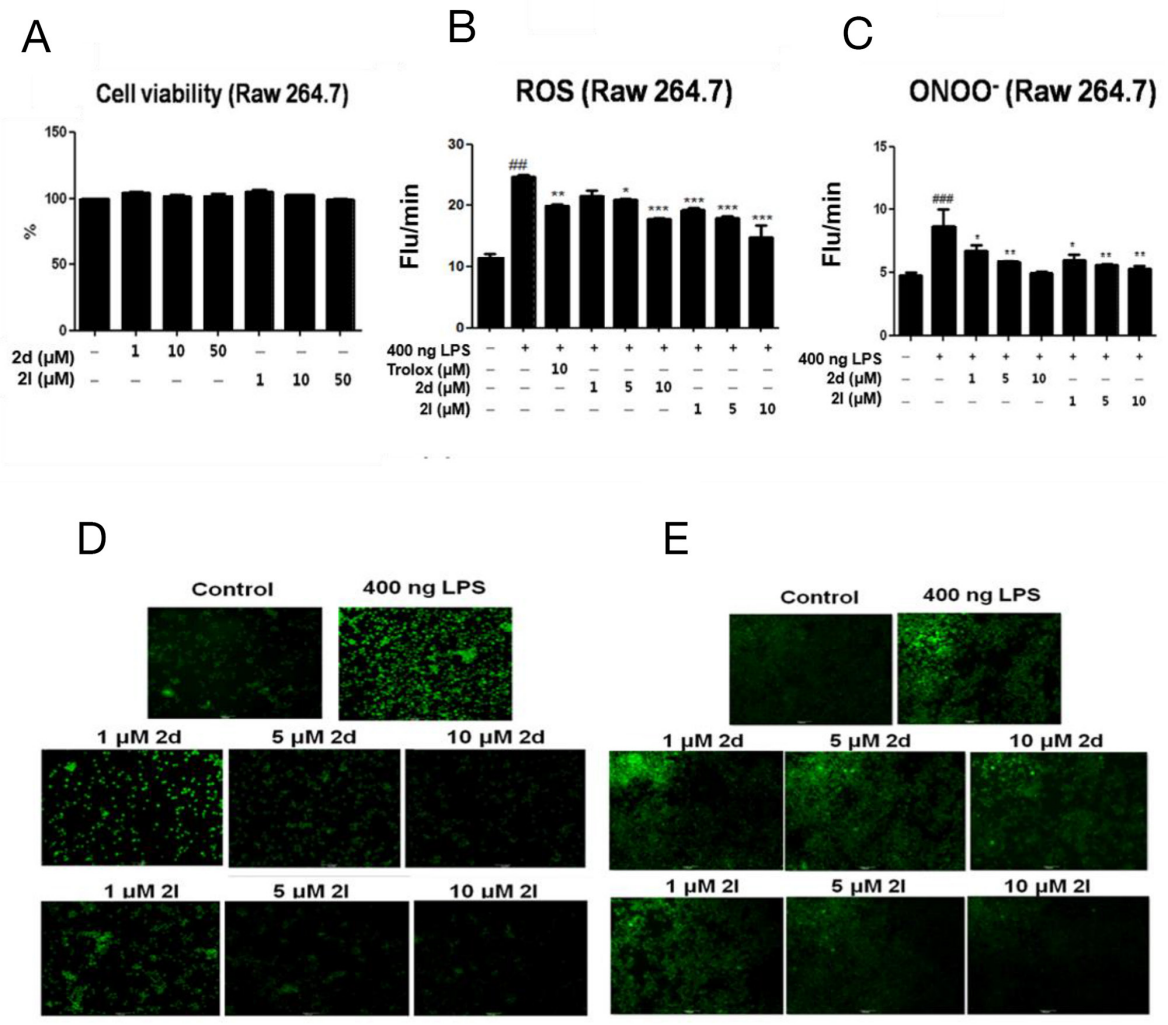
Inhibitory effects of compounds 2d and 2l on LPS-induced oxidative stress in macrophages **(A)** Cell viability was examined inRAW264.7 cells treated with various doses of compounds 2d or 2l (1-50 μM). RAW264.7 cells were pretreated with compounds 2d or 2l (1-10 μM) for 1 h, followed by 400ng LPS treatment for 18 h. Suppression of intracellular **(B)** ROS and **(C)** ONOO^-^ levels by compounds 2d and 2l (1-10 μM). Fluorescence microscopic images show intracellular **(D)** ROS and **(E)** ONOO^-^ in RAW264.7 cells. Values represent the mean ± SEM of three experiments. Data are expressed as % of cell viability. Statistical results of one-way ANOVA followed by the Dunnett’s test: *##p* < 0.01, *###p* < 0.001 vs. non-treatment, ^*^p < 0.05, ^**^p < 0.01, ^***^p < 0.001 vs. LPS-treated cells

Macrophages play an essential role in regulating inflammation in the liver, especially after LPS stimulation [19–21]. We used an RAW264.7 macrophage cell line to investigate the effect of compounds 2d and 2l on LPS-induced oxidative signaling. We confirmed that compounds 2d or 2l were not cytotoxic at the concentration up to 50 μM (Figure 4A). Oxidative stress has been shown to activate macrophages [22]. We investigated whether compounds 2d and 2l exhibited ROS and ONOO^-^ scavenging activity in macrophages. LPS markedly elevated cellular levels of ROS and ONOO^-^ but the compounds dose-dependently lowered these levels in macrophages (Figure 4B-4C). In addition, visual observation of ROS and ONOO^-^ production using fluorescent microscopy showed that compounds 2d and 2l notably reduced ROS and ONOO^-^ generation (Figure 4D-4E), confirming that these compounds suppress LPS-induced oxidative stress in macrophages.

### Effects of compounds 2d and 2l on LPS-induced NF-κB signaling in macrophages

**Figure 5:**
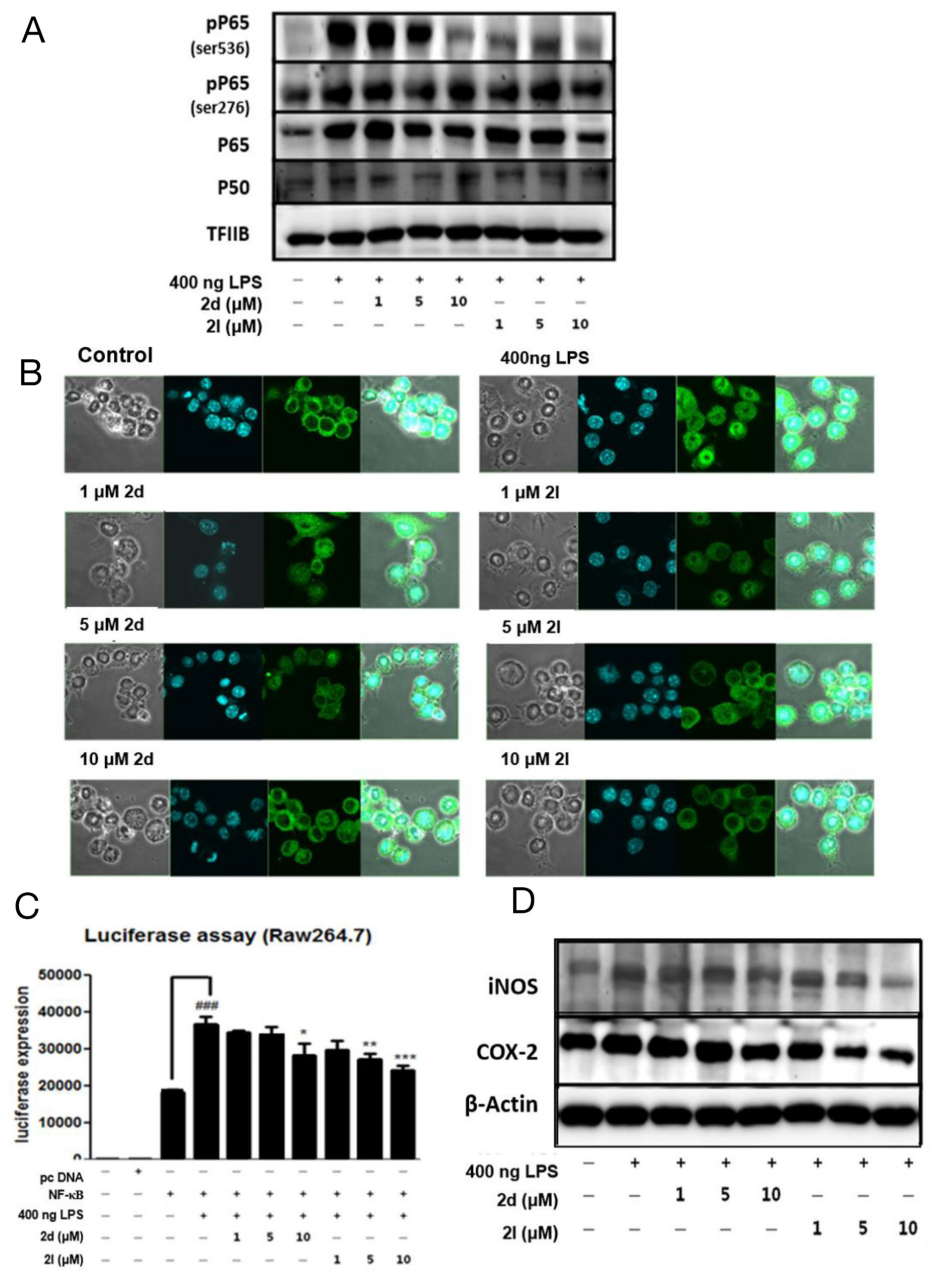
Inhibition of nuclear factor kappa B (NF-κB) activity by compounds 2d and 2l **(A)** RAW264.7 cells were pretreated with compounds 2d or 2l (1-10 μM) for 1 h, followed by 400ng LPS treatment for 2 h. The cells were fractionated into cytosolic and nuclear portions and analyzed by western blot analysis. TFIIB was used as a marker of the nuclear fraction. **(B)** RAW264.7 cells were pretreated with the compound 2d or compound 2l (1-10 μM) for 1 h. After LPS (400ng) was treated for 2 h to induce NF-κB translocation, the cells were stained with an anti-p65 antibody (p65; Green) and Hoechst (nucleus; blue). p65-positive cells were counted and depicted by confocal fluorescence microscopy. The representative images were shown. **(C)** Both compounds 2d and 2l inhibited the transcriptional activity of NF-κB based on a luciferase assay.The NF-kB luciferase reporter vectors were transfected in Raw264.7cells for 12 h. Then, the cells were pretreated with compounds 2d or 2l (1-10 μM) for 1 h, followed by 400ng LPS treatment for 2 h. **(D)** Western blotting was performed to examine the effects of compounds 2d and 2l on cytosolic protein levels of inducible nitric oxide synthase and cyclooxygenase 2 in the liver. β-Actin was used as a loading control in the cytosolic fraction. tatistical results of one-way ANOVA followed by the Dunnett’s test: ^*###*^*p*<0.001 was vs. NF-κB; ^*^p <0.5, ^**^p<0.01, ^***^p<0.001 vs. the LPS/NF-κB-treated group.

The activation of NF-κB signaling is key characteristics of macrophage activation [17]. We examined effects of the compounds on NF-κB activity in macrophages. Western blot analysis showed that phosphorylation of NF-κB at Ser536, was dose-dependently decreased by compounds 2d and 2l in macrophages (Figure 5A). It is known that the NF-κB can be activated through phosphorylation of p65 at Ser536, which is regulated through the toll-like receptor 4- phosphatidylinositol 3 kinase (PI3K) /AKT pathway. We used immunohistochemistry to demonstrate that the compounds inhibited NF-κB translocation. LPS-induced nuclear translocation of NF-κB/p65 was dose-dependently blocked by treatment with compounds 2d and 2l (Figure 5B). To investigate the effect of the compounds on NF-κB transcriptional activity, an NF-κB luciferase assay was performed in macrophages. LPS treatment highly increased NF-κB transcriptional activity, whereas compounds 2d and 2l significantly decreased it (Figure 5C). Likewise, protein levels of the inflammatory proteins inducible nitric oxide synthase and cyclooxygenase 2, downstream targets of NF-κB, were reduced by compounds 2d and 2l (Figure 5D) in macrophages. These data clearly show that the compounds suppress NF-κB activation in macrophages.

### Compounds 2d and 2l inhibit PI3K/AKT signaling in RAW264.7 macrophages

**Figure 6:**
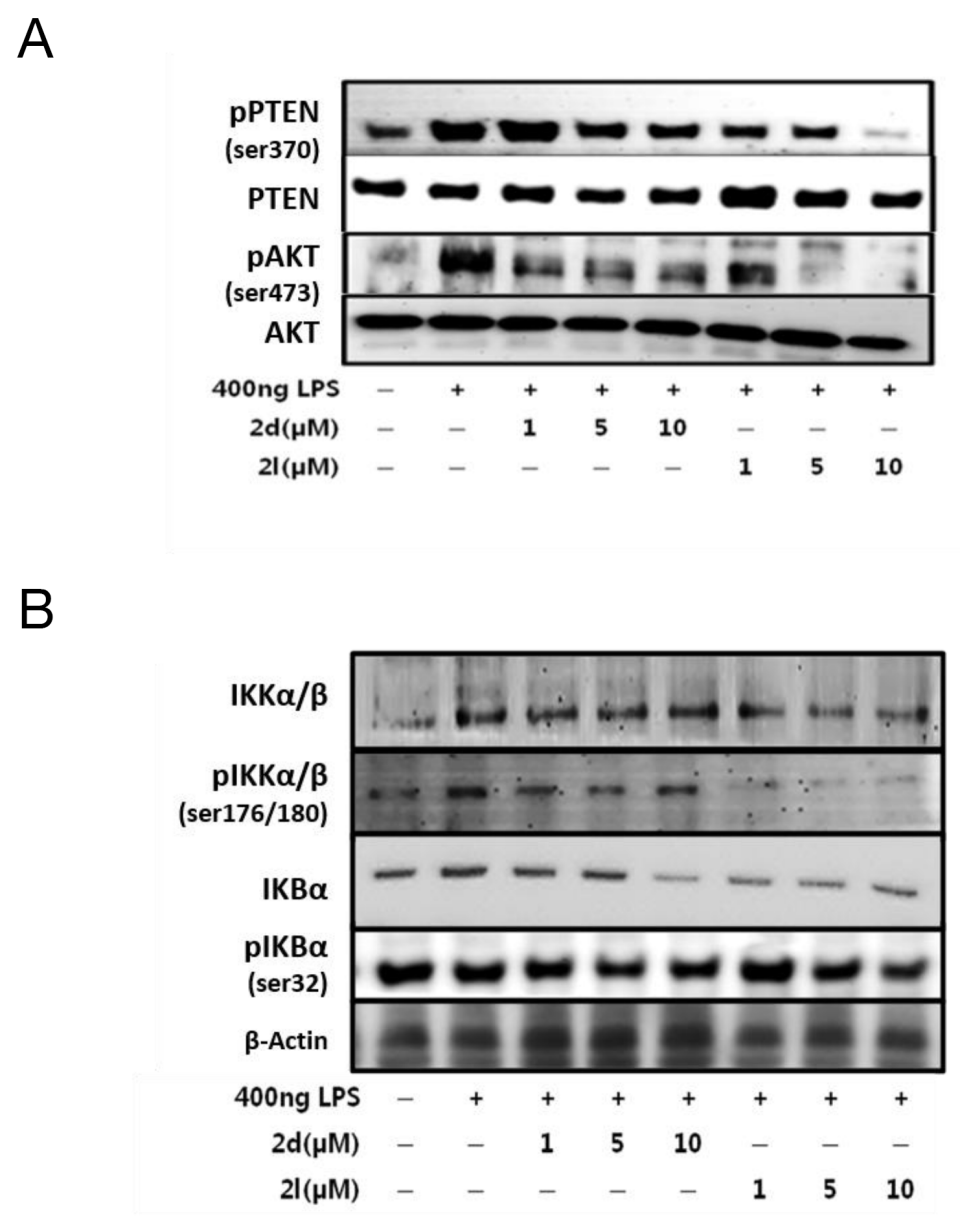
Effects of compounds 2d and 2l on AKT/NF-κB signaling in LPS-stimulated RAW264. 7 cells Western blotting was performed to detect protein levels of **(A)** PTEN and AKT, and **(B)** IKKα/β and IKbα in Raw264.7 cells. β-Actin was used as the loading control.

The phosphatase and tensin homolog deleted on chromosome 10 (PTEN) is an important phosphatase that inhibits the phosphorylation of NF-κB at Ser536 by the PI3K/AKT pathway [23]. Because compounds 2d and 2l inhibited NF-κB phosphorylation at Ser536, we determined by western blot whether these compounds regulated PTEN/AKT signaling. LPS treatment increased phosphorylation PTEN and AKT, but compounds 2d and 2l reduced them (Figure 6A).

AKT phosphorylation activates NF-κB by phosphorylating inhibitor of kappa B kinase (IKK), causing separation of NF-κB from IKK [16]. The treatment of macrophages with LPS for 2 h increased phosphorylation of IKKα/β and inhibitor of kappa B (IκB)α, whereas compounds 2d and 2l inhibited it (Figure 6B). Based on these data, the 2d- and 2l- mediated inhibition of NF-κB activation is likely due to the suppression of PTEN/AKT signaling.

Our study focused on the compounds 2d and 2l based on stronger ROS and ONOO^-^ scavenging activities than other compounds. However, most of the 5-(substituted benzyl)-2-oxo- and 5-(substituted benzyl)-2-thioxo-dihydropyrimidine-4,6(1*H*,5*H*)-dione derivatives significantly decreased SIN-1-induced ROS and ONOO^-^ production. Because the antioxidative effects of compounds 2d and 2l are closely related to the decreased inflammatory signaling, other compounds may also ameliorate inflammation in the liver. Furthermore, it is necessary to examine structural mechanisms by which thio-barbiturate-derived compounds exhibit strong antioxidative activity.

The study showed that the compound 2d- or 2l suppressed NF-κB signaling in macrophages post LPS treatment, but it may not be the only mechanism underlying the compound-mediated decrease in the liver inflammation. Because there are reports that other cell types in the liver including hepatocytes, lymphocytes, dendritic cell, and sinusoidal endothelial cells are also important mediators of inflammation [24]. Thus, it is likely that the anti-inflammatory effects of the compounds 2d and 2l in the liver is not only caused by its effect on macrophages but also arise from its effect on other cell types.

In conclusion, compounds 2d and 2l notably inhibited inflammation triggered by LPS, at least in part, by suppressing PTEN/AKT-mediated NF-κB activation in RAW264.7 cells. We speculate that the anti-inflammatory properties of the compounds are attributed to ROS and ONOO^-^ scavenging activity. Consistently, when administrated orally, compounds 2d and 2l effectively inhibited LPS-induced liver inflammation *in vivo*. Compounds 2d and 2l may be promising pharmacological agents for the treatment of liver inflammatory disorders.

## MATERIALS AND METHODS

### Materials

Compounds 2d and 2l were synthesized in our laboratory. The condensation reaction of the thiobarbituric acid with vanillin (4-hydroxy-3-methoxybenzaldehyde) and syringaldehyde (4-hydroxy-3,5-dimethoxybenzaldehyde) in ethanol and water (1:1) afforded 5-(4-hydroxy-3-methoxybenzylidene)thiobarbituric acid and 5-(4-hydroxy-3,5-dimethoxybenzylidene)thiobarbituric acid, respectively. After ethanol was added to the each product, NaBH_4_ (3.0 eq.) was added portion wise and stirred at room temperature to reduce carbon-carbon double bond of exomethylene of the thiobarbituric acid products. Volatiles were evaporated and water was added to the resulting residue. Precipitates generated after pH adjustment to 7 using 1N-HCl were filtered and washed with ether/water (10 – 20:1) and ethanol/water (10 – 20:1) to give compounds 2d and 2l, respectively. 5-(4-Hydroxy-3-methoxybenzyl)-2-thioxodihydropyrimidine-4,6(1*H*,5*H*)-dione (compound 2d): ^1^H NMR (500 MHz, D_2_O) *δ* 6.76 (s, 1 H), 6.66 (d, 1 H, *J* = 8.0), 6.59 (d, 1 H, *J* = 8.0 Hz), 3.68 (s, 3 H), 3.38 (s, 2 H); ^13^C NMR (100 MHz, D_2_O) *δ* 171.7, 165.2, 147.3, 142.7, 134.4, 120.5, 115.5, 112.4, 94.9, 56.0, 27.2. 5-(4-Hydroxy-3,5-dimethoxybenzyl)-2-thioxodihydropyrimidine-4,6(1*H*,5*H*)-dione (compound 2l): ^1^H NMR (400 MHz, D_2_O) 6.44 (s, 2 H), 3.66 (s, 6 H), 3.37 (s, 2 H); ^13^C NMR (100 MHz, D_2_O) 171.8, 165.2, 147.7, 133.8, 131.8, 105.4, 94.7, 56.4, 27.7. This chemical was dissolved in dimethylsulfoxide (DMSO). Lipopolysaccharide (LPS) (from *Escherichia coli* 0111: B4, CAS registry no. L2630) and other chemical reagents were purchased from Sigma (St. Louis, MO, USA).

### Animal experiments

All animal experiments were approved by the Pusan National University-Institutional Animal Care and Use Committee (PNU-IACUC). C57/BL6 male mice (weight 30-32 g, 6 weeks of age) were purchased from Japan SLC (Hamamatsu, Japan). This study followed the Guide for Animal Care and Use issued by the Institute of Laboratory Animal Resources (ISBN 0-309-05377-3). Fifty C57/BL6 mice were assigned to one of the following six groups: 1) a saline injected group (the control group, *n*=5); 2) a 5 mg/kg LPS group (the LPS group, *n*=5); 3-4) two compound 2d (at 1or 5 μM) plus 5 mg/kg LPS groups (compound 2d+LPS groups, *n*=5); and 5-6) two compound 2l (at 1or 5 μM) plus 5 mg/kg LPS groups (compound 2l+LPS groups, *n*=5). Saline, compound 2d, and compound 2l were administered 2 h prior to LPS (or saline for the control group) treatment. Mice were dissected 2 h after LPS treatment.

### Tissue homogenate

All solutions and materials were maintained at 0 ∼ 4°C. Liver samples were homogenized in 1ml of homogenate buffer A [10 mM KCl, 2 mM MgCl_2_, 1 mM dithiothreitol (DTT), 0.1 mM EDTA, 0.1 mM phenylmethanesulfonyl fluoride (PMSF), 1 μM pepstatin, 2 μM leupeptin, 20 mM β-glycerophosphate, 20 mM NaF, 2 mM Na_3_VO_4_, and 10 mM 4-(2-hydroxyethyl)-1-piperazineethanesulfonic acid (HEPES), pH7.4] with a tissue homogenizer for 20 s and put on ice for 15 min. Then, 125 μl of 10% Nonidet P-40 (NP-40) was added and mixed on ice for 15 s, and the homogenate was centrifuged at 14,000 *g* at 4 °C for 2 min. The supernatants were used as the cytosolic sample. The pellets were resuspended in homogenate buffer C [50 mM KCl, 300 mM NaCl, 0.1 mM PMSF, 10% (v/v) glycerol, 1 μM pepstatin, 2 μM leupeptin, 20 mM β-glycerophosphate, 20 mM NaF, 2 mM Na_3_VO_4_, and 50 mM HEPES,pH 7.8], kept on ice for 30 min, and centrifuged at 14,000 *g* at 4°C for 10 min. Each sample was stored at -80 °C in a deep freezer. Protein concentration was determined using the bicinchoninic acid (BCA) assay using bovine serum albumin (BSA) as a standard.

### Cell culture

RAW264.7 cells (a murine macrophage cell line) were purchased from the American Type Culture Collection (ATCC, Manassas, VA, USA). Cells were used at 37°C in Dulbecco’s Modified Eagle’s Medium (DMEM; Hyclone Laboratories, Logan, Utah, USA) containing 2 mM L-glutamine, 100 units/ml penicillin, 100 μg/ml streptomycin, and 10 % heat-inactivated fetal bovine serum (FBS, Hyclone). Cells were incubated at 37 °C in 5 % CO_2_/ 95% air atmosphere.

### Cell lysis

The cell was harvested at 3,000 rpm at 4 °C for 5 min. The pellets were suspended in lysis buffer containing 10 mM Tris, pH 8.0, 1.5 mM MgCl_2_, 1 mM DTT, 0.1%NP-40, 1 μM pepstatin, and 1 mM *p*-aminobenzamidine on ice for 15 min and then centrifuged at 13,000 *g* at 4 °C for 15 min. The supernatants were used as the cytosolic sample. The pellets were resuspended using nucleus lysis buffer containing 10 mM Tris-pH 8.0, 50 mM KCl, 100 mM NaCl, 1 μM pepstatin, and 1 mM *p*-aminobenzamidine, placed on ice for 30 min, and then centrifuged at 14,000 *g* at 4°C for 30 min. The resultant supernatants were used as the nuclear sample. Protein concentration was determined by the BCA assay using BSA as a standard.

### Cell viability assay

Briefly, RAW264.7 cells were seeded at 1.5 × 10^4^ cells per/well in 96-well plates and then incubated at 37°C for 24 h and treated with samples. Cell viability was measured by the EZ-cy tox cell viability Assay kit (Daeil Lab Service, Seoul, Korea) as previously described.

### Western blot

Western blotting was performed as previously described [25]. Primary antibodies were as follows: pP65 (Ser 536) (sc3020), pP65 (Ser 276) (sc101749), P65 (sc372), COX-2 (cyclooxygenase 2) (sc1747-R), inducible nitric oxide synthase (iNOS) (sc7271), AKT (sc1618), pAKT (Ser 473) (sc7985), inhibitor of kappa B alpha (IKBα) (sc847), pIKBα (Ser32) (sc8404), inhibitor of kappa B kinase alpha/beta (IKKα/β) (sc7607), β-Actin (sc47778), and transcription factor IIB (TFIIB) (sc225) were purchased from Santa Cruz Biotechnology (Dallas, TX, USA). pIKKα/β (Ser 176/180) (#2697s) and PTEN and phosphoinositide dependent kinase 1 (PDK1) Ab sample kit (#9652) were purchased from Cell Signaling (Beverly, MA, USA).

### Quantitation of redox status

To evaluate redox status-scavenging activity, RAW264.7 cells were seeded at 1 × 10^4^ cells/well in a black 96-well plate and incubated overnight with SIN-1 and LPS to induce the generation of reactive oxygen species (ROS). Trolox was used as a positive control as an ROS scavenger, and penicillamine was used as a positive control as a peroxynitrite (ONOO^-^) scavenger.

### ROS measurement

ROS generation was measured using a fluorescence probe, as previously described. Briefly, 50 μM 2′,7′-dichlorodihydrofluorescein diacetate (DCFDA) was added to homogenates for a final volume of 250 μl. Changes in fluorescence were detected with a GENios plate reader (Tecan Instruments, Salzbrug, Austria) with excitation and emission wavelengths set at 485 and 530 nm, respectively. In addition, changes in ROS level were detected using a laser-scanning confocal microscope on RAW264.7 cells seeded at 2 × 10^5^ cells/well in a 6-well cell culture dish.

### ONOO^-^ measurement

ONOO^-^ generation was examined by measuring the oxidation of dihydrorhodamine (DHR123). Briefly, liver homogenate (10 μl) was mixed with the rhodamine solution (50 mM sodium phosphate buffer, 90 mM NaCl, 5 mM diethylene-triamine pentaacetate [DTPA], and DHR 123) to a final volume of 200 μl. Changes in fluorescence intensity were measured every 5 min for 30 min using a fluorescence plate reader at excitation and emission wavelengths of 485 and 530 nm, respectively. In addition, the fluorescence of ONOO− generated in the cytoplasm of RAW264.7 (2 × 10^5^ cells/well) was directly confirmed using a laser-scanning confocal microscope.

### Immunostaining

RAW264.7 cells were seeded at 3 ×10^4^ cells per well in a 12-well plate and incubated overnight. Cells were fixed in 4 % paraformaldehyde solution for 30 min, washed with phosphate buffered saline (PBS), blocked with 3 % normal goat serum (Gibco, Grand Island, NY, USA), and immunostained with rabbit anti-nuclear factor kappa B (NF-κB) antibody (1:500 dilution) at 4 °C overnight. Cells were then washed with Tris-buffered saline (TBS) and incubated for 2 h with anti-rabbit IgG labeled with Alexa Fluor 488 (1:200 dilutions, Invitrogen, Carlsbad, CA, USA). Immunostaining was performed using Hoechst 33342 (1:1,000 dilution, Invitrogen) to visualize the cells, and NF-κB location was determined using confocal laser scanning microscopy (TCS SP2, Leica, Wetzlar, Germany).

### Luciferase assay

For luciferase assays, 1μg of plasmid was transfected into RAW264.7 cells (5×10^4^ cells per well) seeded in a 24-well plate in 500 μL of DMEM supplemented with 10 % FBS at 37 °C in a humidified 95 % air/5 % CO_2_ atmosphere. Cells were transfected with Lipofectamine transfection reagent, and the plasmids used for transfection contained the NF-κB- luciferase reporter vector. After 12 h of transfection, cells were washed and treated with compounds 2d and 2l for 6 h. Luciferase activity was detected using the One-Glo luciferase assay system (Promega, Madison, WI, USA) and measured using a Tecan GENios luminescence plate reader.

### Statistical analysis

All results are expressed as means ± standard error of the means (SEM). One-way analysis of variance (ANOVA) was performed to analyze significant differences among all groups. Dunnett’s test was used to determine the significance between group means. An associated probability (*p-*value) of <0.05 was considered statistically significant.
